# Effect of Graphene Oxide on Mechanical Properties and Durability of Ultra-High-Performance Concrete Prepared from Recycled Sand

**DOI:** 10.3390/nano10091718

**Published:** 2020-08-30

**Authors:** Hongyan Chu, Yu Zhang, Fengjuan Wang, Taotao Feng, Liguo Wang, Danqian Wang

**Affiliations:** 1 College of Civil Engineering, Nanjing Forestry University, Nanjing 210037, China; 2 Jiangsu Key Laboratory of Construction Materials, School of Materials Science and Engineering, Southeast University, Nanjing 211189, China; tgyuzhang@outlook.com (Y.Z.); fengjuan19921118@sina.com (F.W.); taotao_feng@yeah.net (T.F.); wlg_seu@sina.com (L.W.); 3 Advanced and Innovative Materials (AIM) Group, Department of Civil, Environmental and Geomatic Engineering, University College London, London WC1E 6BT, UK; danqian.wang.16@ucl.ac.uk

**Keywords:** ultra-high-performance concrete, mechanical properties, durability, recycled sand, graphene oxide, microstructure

## Abstract

Ultra-high-performance concrete (UHPC) has been used as an advanced construction material in civil engineering because of its excellent mechanical properties and durability. However, with the depletion of the raw material (river sand) used for preparing UHPC, it is imperative to find a replacement material. Recycled sand is an alternative raw material for preparing UHPC, but it degrades the performance. In this study, we investigated the use of graphene oxide (GO) as an additive for enhancing the properties of UHPC prepared from recycled sand. The primary objective was to investigate the effects of GO on the mechanical properties and durability of the UHPC at different concentrations. Additionally, the impact of the GO additive on the microstructure of the UHPC prepared from recycled sand was analysed at different mixing concentrations. The addition of GO resulted in the following: (1) The porosity of the UHPC prepared from recycled sand was reduced by 4.45–11.35%; (2) the compressive strength, flexural strength, splitting tensile strength, and elastic modulus of the UHPC prepared from recycled sand were enhanced by 8.24–16.83%, 11.26–26.62%, 15.63–29.54%, and 5.84–12.25%, respectively; (3) the resistance of the UHPC to penetration of chloride ions increased, and the freeze–thaw resistance improved; (4) the optimum mixing concentration of GO in the UHPC was determined to be 0.05 wt.%, according to a comprehensive analysis of its effects on the microstructure, mechanical properties, and durability of the UHPC. The findings of this study provide important guidance for the utilisation of recycled sand resources.

## 1. Introduction

Ultra-high-performance concrete (UHPC) is a new construction material with excellent mechanical properties and durability [1], which makes it an innovative material in the engineering field [2]. However, its preparation requires high-quality raw materials [3] such as quartz sand or high-grade river sand. With the continuous modernisation and urbanisation progress in China, the domestic construction industry has undergone vigorous development, accompanied by the rapid depletion of raw construction materials, particularly river sand. Currently, there is a shortage of river sand in most parts of China, significantly increasing the price of river sand. In certain areas, there is no river sand at all. In fact, the entire world is facing a shortage of river sand. Therefore, it is critical to find an alternative fine aggregate to replace river sand. With the increasing urbanisation in China, a large amount of construction waste has been generated there in recent years during the processes of construction, reconstruction, expansion, and demolition. In 2017, a total of 2.38 billion tons of construction waste was generated, and this number is expected to exceed 2.6 billion tons by the end of 2020. However, the utilisation rate of construction waste in China is <5% and is significantly lower than that for Japan and Germany [4]. Therefore, transforming construction waste into reclaimed sand and using it as the raw material for preparing UHPC will not only allow the utilisation of construction waste but also turn the waste into a valuable resource that contributes to energy savings and emission reduction. These merits will provide significant social, environmental, and economic benefits.

The preparation of concrete using recycled sand has been widely investigated. Kumar et al. [5] reported that the performance of concrete prepared by using recycled sand remained unaffected when the replacement level of recycled sand was <20 wt.%. Xiao et al. [6] reported that the elastic modulus of concrete will be reduced by 45% if the replacement level of recycled sand is 100%. In general, using recycled sand in concrete degrades its mechanical properties [7,8,9,10,11] and durability [7,8,9,12,13,14,15,16,17]. In the case of concrete prepared from recycled sand, a large interfacial transition layer is formed on the surface of the recycled sand during the hydration process. Additionally, the old mortar formed at the surface of the recycled sand exhibits poor mechanical properties [18]. These features are the primary reasons for the poor mechanical properties of concrete prepared from recycled sand. Compared with normal concrete, concrete prepared using recycled sand has a larger chloride-ion diffusion coefficient [19], a greater carbonation depth [20], and a higher water-absorption rate [21]. These properties indicate that the use of recycled sand degrades the durability of the concrete. Because recycled sand has a high porosity, concrete prepared using recycled sand has a high permeability, eventually degrading its durability. A recent study indicated that the mechanical properties of UHPC deteriorate significantly when the replacement level of recycled sand for river sand exceeds 50% [22]. Thus, it is critical to develop methods that can improve the performance of concrete prepared from recycled sand. Furthermore, few studies have been performed on the preparation of UHPC using recycled sand. Additionally, research on the durability of UHPC prepared from recycled sand is scarce.

The use of nanomaterials can improve the properties of the matrix in cement-based materials prepared from recycled sand, enhancing the material performance. With the advancement of nanotechnology, the mixing of nanomaterials into cement-based materials has been increasingly researched. A wide variety of nanomaterials have been investigated, including nano-silicon oxide [23,24,25], nano-titanium oxide [26], carbon nanotubes [27,28,29], sulfonated graphene [30,31,32], and graphene oxide (GO) [33,34,35,36,37]. GO is a new type of nanomaterial with excellent mechanical properties and good dispersion properties. The elastic modulus and inherent strength of GO are as high as 300 and 112 GPa, respectively [38]. The performance of cement-based materials can be improved via mixing with GO for the following reasons: (1) the filling effect [39], (2) an increasing number of nucleation sites during the hydration of cement [40], and (3) the ability to tune the morphology of cement hydration products [41]. Therefore, the use of GO presents great potential for enhancing the mechanical properties and durability of cement-based materials [33]. Of course, there are some other methods to improve the properties of cement-based materials [42,43]. In particular, nanomaterials have attracted increasing attention, owing to their capability to significantly improve the properties of cement-based materials at a low mixing concentration. However, nanomaterials are expensive, which prevents them from being used in large-scale applications in the field of cement-based materials. With the advancement of nanotechnology, GO can be produced at an industrial scale at low manufacturing costs. Additionally, only an extremely small quantity of GO is required to be mixed in the cement-based material. Consequently, it is economically feasible to use GO for improving the properties of cement-based materials. Thus far, the use of GO to improve the properties of UHPC prepared from recycled sand has not been investigated. Furthermore, most of the previous research focused on the effects of GO on the mechanical properties of concrete made from recycled sand. Few studies have investigated the influence of GO on the concrete durability. Therefore, it is imperative to analyse the effects of GO on both the mechanical properties and durability of UHPC prepared from recycled sand.

In this study, the properties of UHPC prepared from recycled sand were enhanced via mixing with GO additives. The primary objective was to investigate the effects of GO as an additive on the mechanical properties and durability of recycled-sand-based UHPC (RS-UHPC) at different mixing concentrations. Specifically, the effects of the GO additive on the mechanical properties (compressive strength, flexural strength, splitting tensile strength, and elastic modulus) and durability (chloride-ion penetration resistance and freeze–thaw resistance) of the RS-UHPC were systematically analysed at three different concentrations. Additionally, the effects of the GO additive on the microstructure of the RS-UHPC were investigated. The results provide important guidance for the application of UHPC made from recycled sand.

## 2. Experiment

### 2.1. Experimental Materials

**Figure 1:**
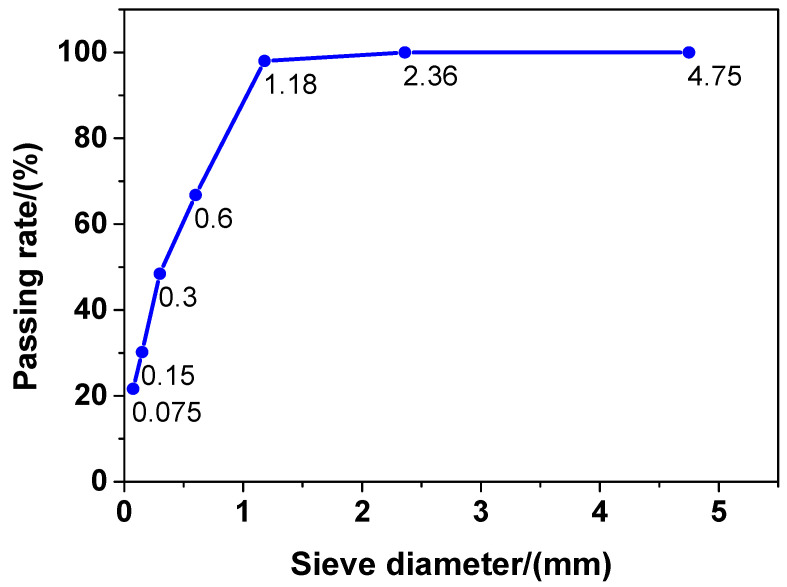
The particle size distribution of recycled sand.

**Figure 2:**
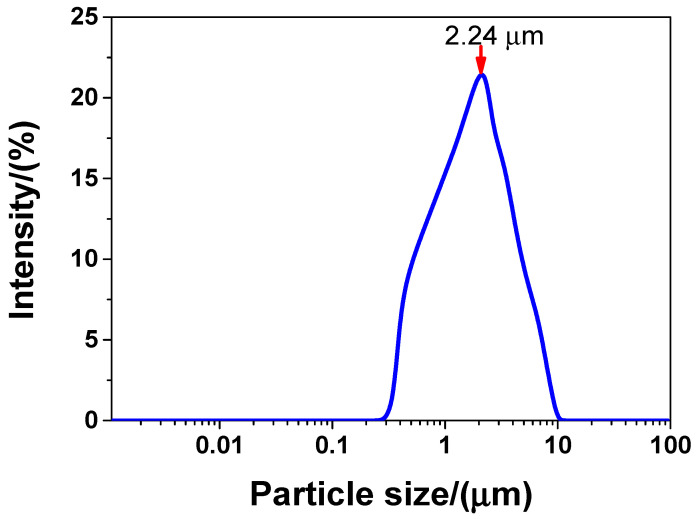
The particle size distribution of GO via dynamic light scattering test.

The following materials were procured to prepare the concrete sample in this study: (1) P·Ⅱ52.5 grade cement produced by Jiangnan-Xiaoyetian Cement Co., Ltd. (Nanjing, China); (2) 95# micro-silicon powder produced by Aiken International Trading (Shanghai) Co., Ltd. (Shanghai, China); and (3) grade Ⅰ fly ash produced by Zhuhai Minghui Trading Co., Ltd. (Zhuhai, China). The chemical compositions of the cement, silicon powder, and fly ash used in this study are presented in Table 1. The recycled sand used in this study was provided by a local construction waste disposal company. The physical properties, grading curve, and chemical composition of the recycled sand are presented in Table 2, Figure 1, and Table 1, respectively. The polycarboxylic acid superplasticiser used in this study was produced by Jiangsu Subote New Materials Co., Ltd. (Nanjing, China). The solid content and water-reducing rate of the superplasticiser were 40% and 33%, respectively. The hooked-end steel fibres used in the experiments were purchased from Aiken International Trading (Shanghai) Co., Ltd. (Shanghai, China). They had a length of 20.0 mm, a diameter of 0.35 mm, and a tensile strength of approximately 3000 MPa. The CaO-type expansion agents were purchased from Jiangsu Subote New Materials Co., Ltd. The chemical composition of the expansion agents is presented in Table 1. Finally, the GO used in this study was produced by Shanghai Carbon Source Huigu New Material Technology Co., Ltd. (Shanghai, China). The physical and chemical properties of the GO are presented in Table 3. The real particle size distribution of liquid GO is shown in Figure 2. As shown in Figure 2, the particle size distribution of the liquid GO was between 0.34 and 9.53 μm, and the mean size was 2.24 μm. The water used in the experiments was tap water.

### 2.2. Mixing Ratio in Experiments

Table 4 presents the mixing ratio of the different components investigated in this study. The initial mixing ratio (labelled as RU0) corresponded to that of RS-UHPC without GO. For the other three mixing ratios, different contents of GO were used in the sample. RU25, RU50, and RU75 labels were used, corresponding to GO contents of 0.025, 0.05, and 0.075 wt.%, respectively. The GO content was evaluated with respect to the mass of the cementing material (mixture of cement and minerals). The steel fibre content in the RS-UHPC sample was fixed at 2.5 vol.% for all the mixing ratios. Different amounts of water reducers were added to the RS-UHPC sample, in order to ensure a consistent working performance among the different sample groups. In particular, the slump expansion was maintained at approximately 560 mm for each group of RS-UHPC samples.

### 2.3. Preparation and Curing of UHPC

Prior to the preparation of the UHPC, the recycled sand was soaked in water for 12 h, fully saturated with water, and then the recycled sand was washed by water, to remove mud. Eventually, the recycled sand was dried naturally in air at ambient temperature. These procedures ensured that the inside of the recycled sand was saturated with water, while the surface was still dry. Subsequently, an ordinary mixer was used to prepare RS-UHPC. The detailed preparation process was as follows. (1) Cement, silica fume, fly ash, and an expansion agent were mixed in the mixer, with stirring for 4 min. (2) Recycled sand was added, and the stirring was continued for 3 min. (3) Approximately ¾ water was added to the water reducer, and thorough mixing was performed, using a glass rod. The product was then added to the mixture obtained in Step (2), followed by 3 min of stirring. (4) The glass rod was rinsed, along with the container of the water reducer with the remaining water. Then, the entire solution was added to the mixture obtained in Step (3). Stirring was continued for 5 min. (5) Steel fibres were added uniformly to the mixture obtained from Step (5), with stirring for 5 min.

Different types of UHPC test pieces were prepared from recycled sand, depending on the objective of the experiment. After the test pieces were moulded, the moulds were wrapped with plastic film and cured for 48 h, at room temperature. Subsequently, the moulds were dissembled, and the RS-UHPC test pieces were further cured in a standard curing room, for 28 days. The temperature and relative humidity of the curing room were maintained at 20 ± 1 °C and >95%, respectively.

### 2.4. Experimental Method

To examine the microstructure of the RS-UHPC, we measured the porosity and pore-size distribution of RS-UHPC samples, using AutoPore IV mercury intrusion porosimetry (Micromeritics Instrument Corporation). This instrument can measure the pore size in the range of 3.6 nm to 400 μm, with a maximum pressure of 414 MPa.

The tensile stress of the RS-UHPC was measured by using a universal testing machine at a loading rate of 0.80 MPa/s. The dimensions of the test piece were 100 × 100 × 100 mm^3^. There were eight RS-UHPC test pieces for each component mixing ratio. The tensile strength of the RS-UHPC was determined as the average of five experimental measurements. The flexural strength of the RS-UHPC was measured, using a universal testing machine and a four-point flexural experimental device at a loading rate of 0.08 MPa/s. The dimensions of the test piece were 100 × 100 × 400 mm^3^. There were 10 RS-UHPC test pieces for each component mixing ratio. The flexural strength of the RS-UHPC was determined by taking the average measurements for five splitting-tensile-strength experiments. The splitting tensile strength of the RS-UHPC was measured by using a universal testing machine and a splitting tensile strength experimental device at a loading rate of 0.08 MPa/s. The dimensions of the test piece were 100 × 100 × 100 mm^3^. Eight RS-UHPC test pieces were prepared for each component mixing ratio. The splitting tensile strength of the RS-UHPC was determined as the average of five experimental measurements. Finally, the elastic modulus of the RS-UHPC was measured, using a universal testing machine and a micro-deformation experimental device at a loading rate of 0.08 MPa/s. The dimensions of the test piece were 100 × 100 × 300 mm^3^. There were 10 RS-UHPC test pieces for each component mixing ratio. The elastic modulus of the RS-UHPC was determined as the average of six experimental measurements. All four mechanical properties of the RS-UHPC were measured in accordance with the Chinese standard “GB/T, 50081-2002” [44].

The chloride-ion penetration resistance and freeze–thaw resistance of the RS-UHPC were analysed in accordance with the Chinese standard “GB/T 50082-2009” [45]. The test pieces used for the durability test were first cured under standard curing conditions, for 91 days. The microstructure of the RS-UHPC became more stable during this curing process. The chloride-ion permeability of the RS-UHPC was characterised, using the rapid chloride-ion migration coefficient method. Such a test requires a test piece with a diameter of 100 mm and a height of 50 mm. The frost resistance of the RS-UHPC was characterised via a rapid freeze–thaw experiment. The dimensions of the test piece were 100 × 100 × 100 mm^3^. The mass and dynamic elastic modulus of the test piece were measured prior to the freeze–thaw experiment and every 30 test cycles during the experiment. A total of 10 RS-UHPC test pieces were prepared for the durability test. The experiments were performed five times, and the average values of the measurements were recorded.

## 3. Results and Discussion

### 3.1. Microstructure

**Figure 3:**
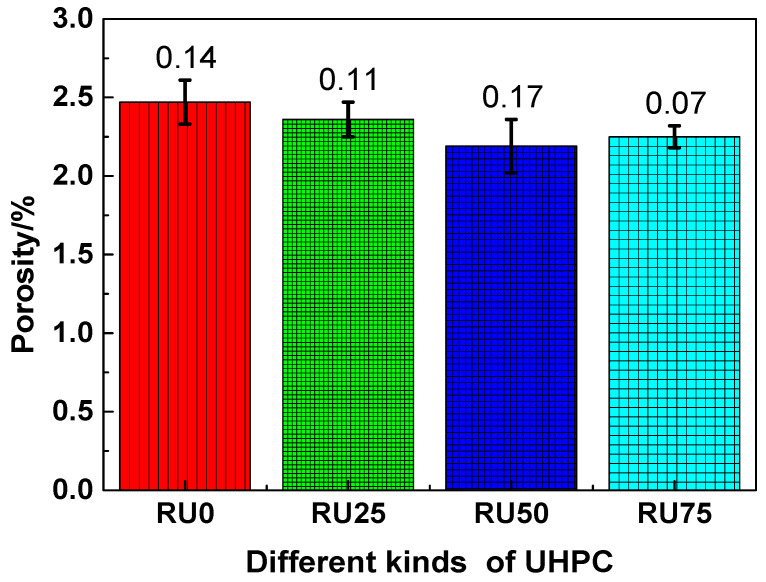
The porosity of RS-UHPC with different contents of GO.

The porosity of the RS-UHPC samples containing different concentrations of GO is shown in Figure 3. The porosities of all the RS-UHPC samples containing GO were lower than those of the samples without GO. This suggests that the addition of GO can reduce the porosity of RS-UHPC. The porosities of RU0, RU25, RU50, and RU75 were measured to be 2.47%, 2.36%, 2.19%, and 2.25%, respectively. The porosities of these samples decreased in the following order: RU0 > RU25 > RU75 > RU50. These values indicate that an increasing GO content leads to a nonlinear reduction in the porosity of RS-UHPC. The porosity of the RS-UHPC prepared in this study was lower than those of low-heat concrete [46], sacrificial concrete [47,48], and UHPC mixed with coarse aggregates [49]. The lowest porosity of RS-UHPC was observed when the GO concentration was 0.05 wt.% Therefore, from the perspective of the porosity, the optimum GO concentration to be mixed in RS-UHPC is 0.05 wt.%. The porosities of RU25, RU50, and RU75 were lower than that of RU0 by 4.45%, 11.34%, and 8.91%, respectively. This suggests that adding GO can improve the pore structure of RS-UHPC. Because GO can enhance the degree of cement hydration [50], adding GO increases the amount of hydration products in cement-based materials. Furthermore, the addition of GO provides a denser interfacial transition layer in cement-based materials [51]. These features result in a reduced porosity in RS-UHPC samples.

**Figure 4:**
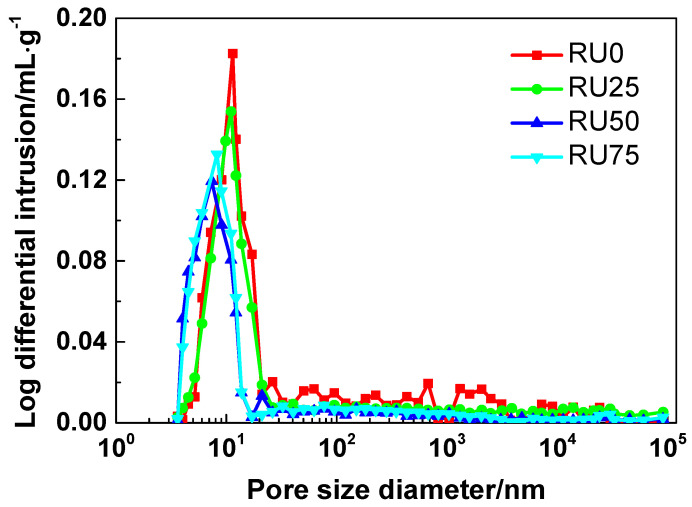
The pore-size distribution of RS-UHPC with different contents of GO.

The pore-size distributions of RS-UHPC containing different concentrations of GO are shown in Figure 4. Different RS-UHPC samples exhibited similar pore-size distribution patterns. A typical peak was observed in the pore-size distribution curve for all the RS-UHPC samples. This peak corresponded to the most probable pore size. As indicated by the results, the most probable pore size was 11.42, 11.04, 7.38, and 8.23 nm for RU0, RU25, RU50, and RU75, respectively. The most probable pore sizes decreased in the order of RU0 > RU25 > RU75 > RU50, in agreement with the porosity measurements. Soliman and Tagnit-Hamou [52] reported that the most probable pore size of UHPC is approximately 10 nm, which is also consistent with our experimental finding. Additionally, the most probable pore sizes of RU25, RU50, and RU75 are all smaller than that of RU0. This behaviour indicates that the pore structure of RS-UHPC can be improved by adding GO.

### 3.2. Mechanical Properties

#### 3.2.1. Compressive Strength

**Figure 5:**
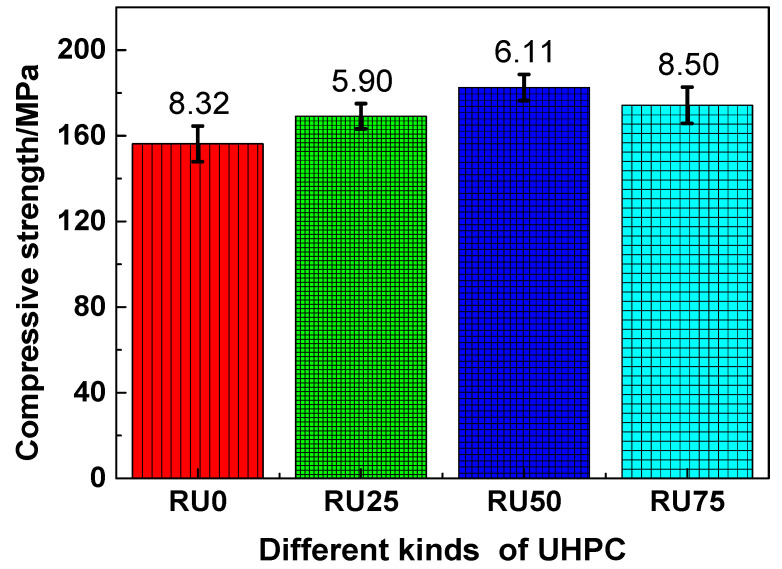
The compressive strength of RS-UHPC with different contents of GO.

The compressive strengths of the RS-UHPC samples containing different concentrations of GO are presented in Figure 5. As shown, the RS-UHPC samples containing GO generally had a higher compressive strength than those without GO. This suggests that adding GO can improve the compressive performance of RS-UHPC. Specifically, the compressive strengths of RU0, RU25, RU50, and RU75 were measured to be 156.21, 169.08, 182.50, and 174.23 MPa, respectively. The compressive strength of RU0 was higher than the minimum strength requirement of 150 MPa for UHPC. Thus, from the perspective of the mechanical properties, recycled sand can be used as the raw material for preparing UHPC. The compressive strengths of RU25, RU50, and RU75 were 8.24%, 16.83%, and 11.54% higher, respectively, than that of RU0. These results indicate that the compressive strength of UHPC does not increase linearly with the increasing GO concentration. In contrast, there exists an optimum GO concentration at which the optimal mechanical properties are achieved. From the perspective of the tensile strength, the optimum concentration of GO to be mixed in RS-UHPC is 0.05 wt.%. In this study, the enhancement in the tensile strength of RS-UHPC was attributed to the following two factors: (1) The presence of GO can enhance the degree of hydration of the cement-based material and improve its microstructure [50], and (2) adding GO can change the hydration products of cement [53].

#### 3.2.2. Flexural Strength

**Figure 6:**
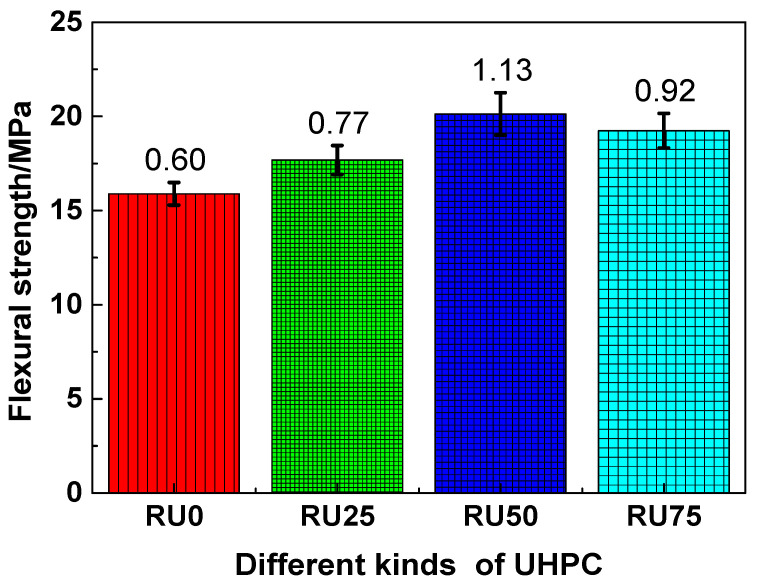
The flexural strength RS-UHPC with different contents of GO.

The flexural strengths of RS-UHPC samples containing different concentrations of GO are shown in Figure 6. Similar to the trend observed for the compressive strength, the flexural strengths of RS-UHPC samples containing GO were generally higher than those without GO. This indicates that adding GO can improve the flexural strength of RS-UHPC. The flexural strengths of RU0, RU25, RU50, and RU75 were 15.89, 17.68, 20.12, and 19.24 MPa, respectively. Thus, the flexural strengths decreased in the order of RU50 > RU75 > RU25 > RU0. The flexural strengths of RU25, RU50, and RU75 were improved by 11.26%, 26.62%, and 21.08%, respectively, compared with that of RU0. The results indicate that the flexural strength of RS-UHPC does not increase linearly with the increasing GO concentration. The flexural strength of the RS-UHPC was maximised at a GO concentration of 0.05 wt.%. Therefore, the optimum concentration of GO to be mixed in RS-UHPC is 0.05 wt.%, from the perspective of the flexural strength. In this study, the flexural strength of the GO-enhanced RS-UHPC ranged from 15.89 to 20.12 MPa for the different GO concentrations. This flexural strength exceeds that of previously reported UHPC prepared from river sand [54]. The flexural strength of UHPC is primarily affected by the following factors: the degree of hydration of the cement, the mechanical strength of the slurry, the bonding strength between the steel fibres and the matrix, and the dispersion performance of the steel fibres [55,56]. Considering that the presence of GO can enhance the degree of cement hydration and improve the microstructure of the cement-based material [50], adding GO to RS-UHPC can improve its flexural strength.

#### 3.2.3. Splitting Tensile Strength

**Figure 7:**
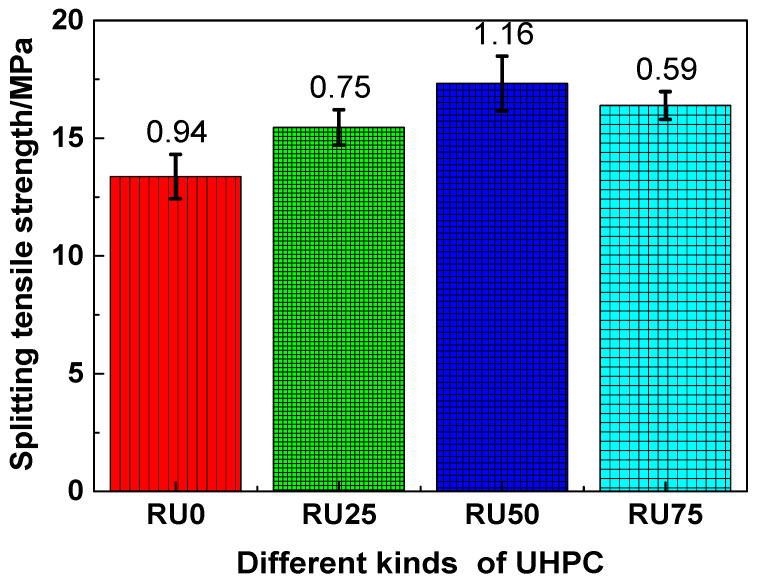
The splitting tensile strength of RS-UHPC with different contents of GO.

The splitting tensile strengths of RS-UHPC samples containing different concentrations of GO are shown in Figure 7. Similar to the trends observed for the compressive strength and flexural strength, the splitting tensile strengths of the RS-UHPC samples containing GO were generally higher than those of the samples without GO. This suggests that adding GO can improve the splitting tensile strength of RS-UHPC. Specifically, the splitting tensile strengths of RU0, RU25, RU50, and RU75 were 13.37, 15.46, 17.32, and 16.39 MPa, respectively. Thus, the splitting tensile strengths decreased in the order of RU50 > RU75 > RU25 > RU0. The splitting tensile strengths of RU25, RU50, and RU75 were improved by 15.63%, 29.54%, and 22.59%, respectively, compared with that of RU0. These values suggest that the splitting tensile strength of RS-UHPC does not increase linearly with the increasing GO concentration. The splitting tensile strength of the RS-UHPC was maximised at a GO concentration of 0.05 wt.%. Therefore, the optimum concentration of GO to be mixed in RS-UHPC is 0.05 wt.%, from the perspective of the splitting tensile strength. The study of Wang et al. [57] revealed that the splitting tensile strength of concrete made from recycled sand depends on the strength of the new mortar, the strength of the recycled aggregate, and the bonding strength of these two components in the concrete. Adding GO can increase the degree of cement hydration and improve the microstructure of cement-based materials [50]. Therefore, adding GO to RS-UHPC can improve the strength of new mortar in the concrete and enhance its splitting tensile strength.

#### 3.2.4. Elastic Modulus

**Figure 8:**
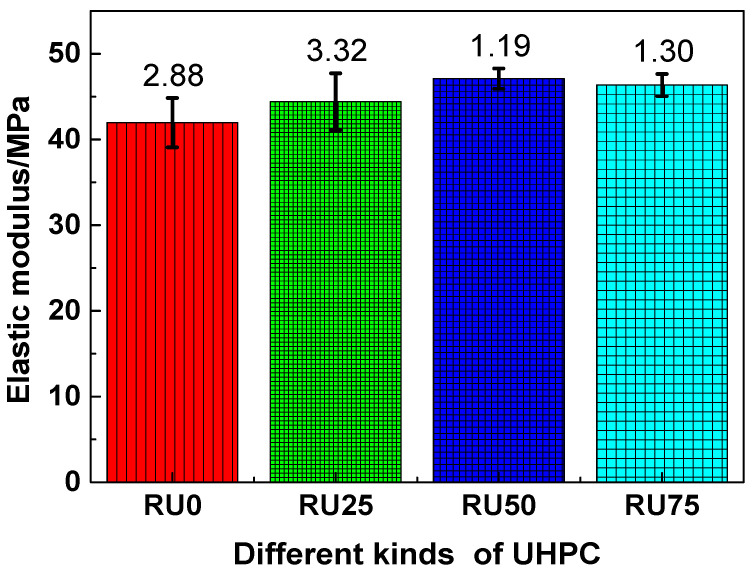
The elastic modulus of RS-UHPC with different contents of GO.

The elastic moduli of the RS-UHPC samples containing different concentrations of GO are shown in Figure 8. Similar to the trends observed for the tensile strength, flexural strength, and splitting tensile strength, the elastic moduli of the RS-UHPC samples containing GO were generally higher than those of the samples without GO. This suggests that adding GO can improve the elastic modulus of RS-UHPC. The elastic moduli of RU0, RU25, RU50, and RU75 were 41.96, 44.41, 47.10, and 46.35 GPa, respectively. Thus, the elastic moduli decreased in the order of RU50 > RU75 > RU25 > RU0. The elastic moduli of RU25, RU50, and RU75 were improved by 5.84%, 12.25%, and 10.46%, respectively, compared with that of RU0. These values suggest that the elastic modulus of RS-UHPC does not increase linearly with the increasing GO concentration. The elastic modulus of the RS-UHPC was maximised at a GO concentration of 0.05 wt.%. Therefore, the optimum concentration of GO to be mixed in RS-UHPC is 0.05 wt.%, from the perspective of the elastic modulus. The elastic modulus of the RS-UHPC sample prepared in this study ranged from 41.96 to 47.10 GPa, which exceeds the value for UHPC prepared from recycled sand [58] and is comparable to that for UHPC prepared from river sand [59], but is slightly lower than that of UHPC produced by Aeolian sand [60]. Ordinary concrete is generally composed of aggregates, a cement slurry, and an interface transition zone. The elastic modulus of concrete mainly depends on the elastic moduli of these three components [61]. The addition of GO can improve the degree of cement hydration and enhance the microstructure of cement-based materials [50]. Furthermore, the addition of GO can provide a denser interfacial transition layer in cement-based materials [51]. Therefore, adding GO can enhance the resistance of RS-UHPC to elastic deformation.

As indicated by the aforementioned results, adding GO to the UHPC sample at a concentration of 0.075 wt.% (higher than the optimum GO content) yielded a slight degradation in the mechanical properties, as compared with the optimum level. This is consistent with the results of Wang et al. [62]. It can be seen from Figure 3 and Figure 4 that the porosity and the most probable pore size of RU75 were higher than that of RU50, respectively, suggesting that the microstructure of RU75 was worse than that of RU50. When the GO is mixed in UHPC at a high concentration, the large specific surface area and the strong intermolecular forces (e.g., van der Waals force) associated with GO cause it to agglomerate. Consequently, the GO becomes less dispersed in the RS-UHPC. Therefore, adding GO to UHPC at a concentration higher than the optimum value slightly degrades all the mechanical properties of UHPC.

### 3.3. Durability

#### 3.3.1. Chloride-Ion Penetration Resistance

**Figure 9:**
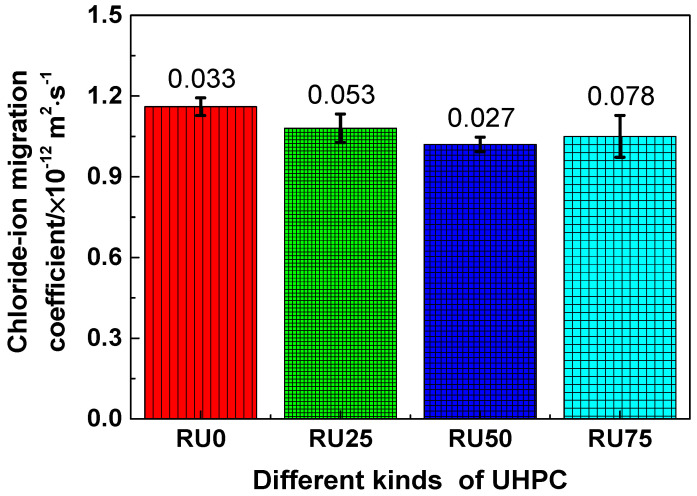
The chloride-ion migration coefficient of RS-UHPC with different contents of GO.

The chloride-ion penetration resistance of RS-UHPC samples containing different concentrations of GO is shown in Figure 9. Generally, the chloride-ion migration coefficients of the RS-UHPC samples containing GO were smaller than those of the RS-UHPC samples without GO. This indicates that adding GO can reduce the chloride-ion migration coefficient of RS-UHPC. The chloride-ion migration coefficients of RU0, RU25, RU50, and RU75 were 1.16 × 10^−12^, 1.08 × 10^−12^, 1.02 × 10^−12^, and 1.05 × 10^−12^ m^2^ s^−1^, respectively. Thus, the migration coefficients decreased in the order of RU0 > RU25 > RU75 > RU50. This sequence suggests that the chloride-ion migration coefficient of UHPC does not decrease linearly with the increasing GO concentration. The chloride-ion migration coefficients of RU25, RU50, and RU75 were reduced by 6.90%, 12.07%, and 9.48%, respectively, compared with that of RU0. Therefore, adding GO to RS-UHPC can significantly improve its chloride-ion penetration resistance. This finding is consistent with the results of Guo et al. [63]. The chloride-ion migration coefficient of the RS-UHPC was minimised when GO was mixed in the UHPC at a concentration of 0.05 wt.%. Thus, the optimum concentration of GO to be mixed in RS-UHPC is 0.05 wt.%, from the perspective of the chloride-ion migration coefficient. The chloride-ion migration coefficient of the RS-UHPC sample prepared in this study is significantly smaller than that previously reported for UHPC [64]. Because GO can enhance the pore structure [65] and improve the microstructure [50] of cement-based materials, the number of chloride ions transported through the capillary channels is reduced with the addition of GO. Furthermore, adding GO can improve the pore structure and volume stability of the cement-based material; accordingly, the number of large pores in the cement-based material is reduced with the introduction of GO. Consequently, the migration of chloride ions is impeded by GO. In summary, adding GO to RS-UHPC improves its resistance to chloride-ion penetration.

#### 3.3.2. Freeze–Thaw Resistance

**Figure 10:**
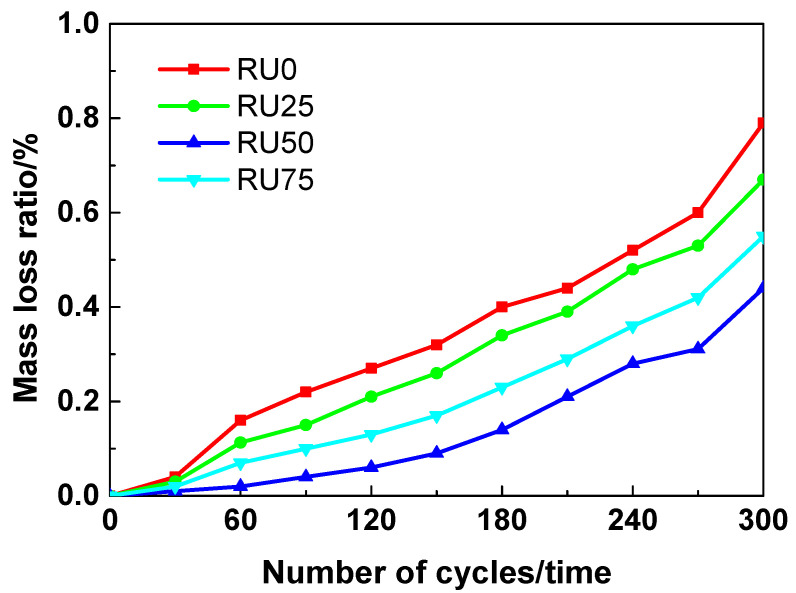
The mass loss ratio of RS-UHPC with different contents of GO.

The mass loss rates of RS-UHPC samples containing different concentrations of GO are shown in Figure 10. Increasing the number of freeze–thaw cycles resulted in a higher mass loss rate for all the different types of RS-UHPC samples. Generally, the mass loss rate of RS-UHPC containing GO was lower than that of UHPC without GO. This suggests that GO can improve the freeze–thaw resistance of RS-UHPC. The mass loss rates of the RS-UHPC samples prepared in this study decreased in the following order: RU0 > RU25 > RU75 > RU50. The mass loss rate of the RS-UHPC was minimised when GO was mixed in the UHPC at a concentration of 0.05 wt.%. Therefore, the optimum concentration of GO to be mixed in RS-UHPC is 0.05 wt.%, from the perspective of the mass loss rate. The mass loss rates of RU0, RU25, RU50, and RU75 after 300 freeze–thaw test cycles were 0.79%, 0.67%, 0.44%, and 0.55%, respectively. As indicated by these results, the mass loss of the RS-UHPC prepared in this study during the 300 freeze–thaw test cycles was almost negligible. This finding is consistent with previously reported freeze–thaw experimental results for ordinary UHPC [64].

**Figure 11:**
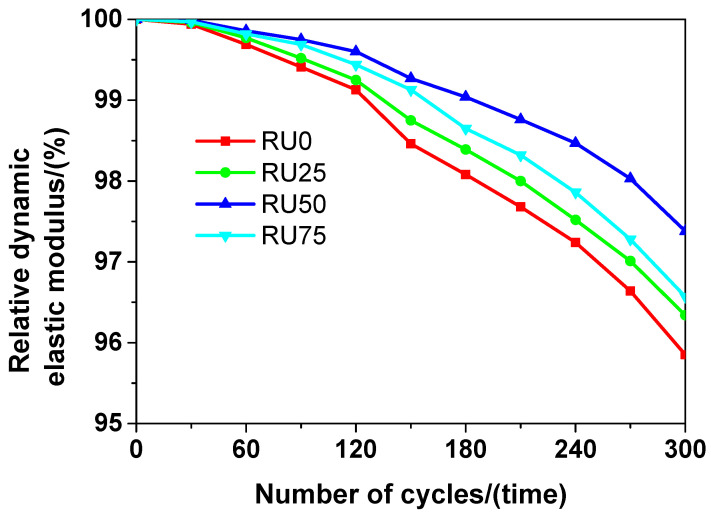
The relative dynamic elastic modulus of RS-UHPC with different contents of GO.

The relative dynamic elastic moduli of the RS-UHPC samples containing different concentrations of GO are shown in Figure 11. The relative dynamic elastic modulus of the RS-UHPC samples decreased with the increasing number of freeze–thaw cycles. Generally, the relative dynamic elastic modulus of the RS-UHPC containing GO was higher than that of the RS-UHPC without GO. Thus, adding GO to RS-UHPC can improve its freeze–thaw resistance. The relative dynamic elastic moduli of the RS-UHPC samples prepared in this study decreased in the following order: RU50 > RU75 > RU25 > RU0. These results indicate that the relative dynamic elastic modulus of the RS-UHPC was maximised at a GO concentration of 0.05 wt.%. Thus, the optimum concentration of GO to be mixed in RS-UHPC is 0.05 wt.%, from the perspective of the relative dynamic elastic modulus. After 300 freeze–thaw test cycles, the relative dynamic elastic moduli of RU0, RU25, RU50, and RU75 were 95.85%, 96.34%, 97.38%, and 96.57%, respectively. These results indicate that the reduction in the relative dynamic elastic modulus was almost negligible for the RS-UHPC after 300 freeze–thaw test cycles, which is consistent with the results of Karim et al. [64].

According to the previously reported criterion [45], the freeze–thaw cycling test was terminated once either of the following two conditions was satisfied: (1) the total mass loss rate reached 5%, and (2) the relative dynamic elastic modulus was reduced to <60% of the initial value. As indicated by the measurements collected in this study, neither of the conditions was met for RS-UHPC during the 300 freeze–thaw test cycles. Therefore, the RS-UHPC prepared in this study had excellent freeze–thaw resistance. Because the presence of GO can provide a denser microstructure and interfacial transition zone in cement-based materials [50,51], adding GO to RS-UHPC can improve its freeze–thaw resistance. Furthermore, GO can enhance the pore structure of cement-based materials [65]. Thus, the water transport in RS-UHPC is impeded during freeze–thaw cycling after the addition of GO. Hence, adding GO to RS-UHPC can improve its freeze–thaw resistance.

The chloride-ion penetration resistance and freeze–thaw resistance of the RS-UHPC were both degraded slightly when GO was mixed in the UHPC at a concentration of 0.075 wt.% (exceeding the optimum concentration). This performance degradation is attributed to the same factors responsible for the degradation of the mechanical properties, i.e., adding GO to UHPC at a high concentration results in the agglomeration of GO.

In summary, the RS-UHPC samples prepared in this study had excellent mechanical properties and durability. According to a comprehensive analysis of the microstructure, mechanical properties, and durability of UHPC, the optimum concentration of GO to be mixed in UHPC was determined to be 0.05 wt.%. The results indicated that adding GO can enhance the pore structure of RS-UHPC, which was a major factor contributing to the improved mechanical properties and durability of the RS-UHPC prepared in this study.

It should be highlighted that the mechanical properties and durability of RS-UHPC were significantly enhanced due to the incorporation of a small quantity of GO, because the degree of cement hydration of RS-UHPC was increased, and the pore structure of RS-UHPC was improved. However, the mechanical properties and durability of RS-UHPC were slightly decreased, when the GO exceeded the optimum concentration, because of the poor ability of GO to be dispersed in RS-UHPC. The optimum concentration of GO to be mixed in RS-UHPC was determined to be 0.05 wt.%, considering the effects of GO on the microstructure, mechanical properties, and durability of RS-UHPC.

## 4. Conclusions

A systematic study was performed to analyse the effects of GO additives on the mechanical properties and durability of RS-UHPC at different mixing concentrations. Furthermore, the effects of GO on the microstructure of RS-UHPC were investigated. According to the results, the following major conclusions are drawn.

(1) The porosity of the RS-UHPC ranged between 2.19% and 2.47%. Adding GO to the RS-UHPC reduced the porosity by 4.45–11.34%. The most probable pore size of the RS-UHPC was reduced with the GO addition. These findings indicate that adding GO to RS-UHPC can improve its pore structure.
(2) The tensile strength of the RS-UHPC ranged between 156.21 and 182.50 MPa. Adding GO to the RS-UHPC enhanced its tensile strength by 8.24–16.83%. This result indicates that adding GO to RS-UHPC can improve its tensile strength.
(3) The flexural strength of the RS-UHPC ranged between 15.89 and 20.12 MPa. Adding GO to the RS-UHPC enhanced its flexural strength by 11.26–26.62%. This result suggests that adding GO to RS-UHPC can improve its flexural strength.
(4) The splitting tensile strength of the RS-UHPC ranged between 13.37 and 17.32 MPa. Adding GO to the RS-UHPC enhanced its splitting tensile strength by 15.63–29.54%. This result indicates that adding GO to RS-UHPC can improve its splitting tensile strength.
(5) The elastic modulus of the RS-UHPC ranged between 41.96 and 47.10 GPa. Adding GO to RS-UHPC increased its elastic modulus by 5.84–12.25%. This result suggests that adding GO to RS-UHPC can enhance its resistance to elastic deformation.
(6) The chloride-ion migration coefficient of the RS-UHPC ranged between 1.02 × 10^−12^ and 1.16 × 10^−12^ m^2^·s^−1^. Adding GO to the RS-UHPC reduced its chloride-ion migration coefficient by 6.90–12.07%. This result indicates that adding GO to RS-UHPC can improve its resistance to chloride-ion penetration.
(7) The mass loss rate and relative dynamic elastic modulus of the RS-UHPC after 300 freeze–thaw test cycles were approximately 0.44–0.79% and 95.85–97.38%, respectively. These results indicate that adding GO to RS-UHPC can improve its freeze–thaw resistance.
(8) According to the comprehensive analysis of the effects of GO on the microstructure, mechanical properties, and durability of RS-UHPC, the optimum concentration of GO to be mixed in RS-UHPC was determined to be 0.05 wt.%.

## Figures and Tables

**Table 1:** The chemical compositions of cement, fly ash, silica fume, recycled sand, and expansive agent (wt.%).

Chemical Composition	Cement	Fly Ash	Silica Fume	Recycled Sand	Expansive Agent
CaO	64.78	18.93	0.22	20.95	68.21
Al_2_O_3_	5.92	7.26	0.39	14.32	7.46
SiO_2_	20.45	73.15	96.21	55.09	6.37
Fe_2_O_3_	3.13	0.09	0.65	3.48	4.15
MgO	1.42	0.17	0.17	1.69	1.25
SO_3_	3.07			1.47	12.56
K_2_O	0.74	0.06		1.60	
Na_2_O	0.18	0.22		0.65	
TiO_2_	0.31	0.05		0.54	
P_2_O_5_		0.07		0.21	

**Table 2:** The physical properties of the fine recycled sand.

Crushing Index (%)	Apparent Density (kg/m^3^)	Water Absorption (%)	Fineness Modulus
22.6	2548	6.13	2.45

**Table 3:** The physical and chemical properties of graphene oxide (GO).

Purity	Thickness (nm)	Flake Diameter (μm)	Carbon Content (%)	Oxygen Content (%)	Dispersant
98%	~1	0.2–10	~46	~53	water

**Table 4:** The mix proportions of recycled-sand-based ultra-high-performance concrete (RS-UHPC) (kg/m^3^).

Mixture	RU0	RU25	RU50	RU75
Cement	690	690	690	690
Fly ash	345	345	345	345
Silica fume	115	115	115	115
Recycled sand	1265	1265	1265	1265
Expansive agent	34.5	34.5	34.5	34.5
Steel fibre	195	195	195	195
Water	207	207	207	207
Water reducer	23	24.6	29.4	36.1
GO	0	0.2875	0.5750	0.8625
